# DIETARY INFLAMMATION AND FALL RISK IN THE ELDERLY: DII AND E-DII SCORE ANALYSIS FROM THE HEALTH AND RETIREMENT STUDY

**DOI:** 10.1093/geroni/igae098.2832

**Published:** 2024-12-31

**Authors:** Afsaneh Fallahi, Matthew Lohman, James Hebert, Sherry Price

**Affiliations:** University of South Carolina, New York, New York, United States; University of South Carolina, Columbia, South Carolina, United States; University of South Carolina, Columbia, South Carolina, United States; University of South Carolina, Columbia, South Carolina, United States

## Abstract

**Background and Objective:**

The Dietary Inflammatory Index (DII) and Energy-adjusted DII (E-DII) are quantitative tools assessing the inflammatory potential of a diet, but these metrics have seldom been applied or compared in a large representative population of older adults. This study aims to calculate and evaluate the association of these indices with fall risk in the elderly.

**Methods:**

DII and E-DII scores were computed for a subsample of 8,035 respondents from the 2013 Health Care and Nutrition Study (HCNS), integrated within the Health and Retirement Study (HRS). Using self-reported data from the Harvard FFQ for 33 out of 45 nutrient parameters, individual DII, and E-DII (adjusted per 1000 kcal intake) scores were computed. Using 2022 HRS data on the history of falls, which was measured binary, and after adjusting for other covariates, logistic regression analyses in SAS software were conducted to assess the associations of interest.

**Results:**

The mean DII score was 0.56 (SD = 2.74) and the E-DII was -0.08 (SD = 2.01). The DII model showed no significant association with fall risk (F-value: 0.85, p = 0.36). Conversely, the E-DII model demonstrated a significant inverse association (F-value: 10.49, p= 0.0012) with an E-DII coefficient of -0.059, p = 0.00012.

**Conclusion:**

Results suggest that higher anti-inflammatory diets, according to E-DII scores, are associated with a lower fall risk among the elderly. E-DII may better reflect the diet’s impact on health outcomes. Keywords: DII, E-DII, HRS, Falls, Older Adults

